# Proceedings of the Terrell Medical Society

**Published:** 1888-07

**Authors:** 


### PROCEEDINGS OF THE TERRELL MEDICAL SOCIETY.

Dr. L. M. Stroud in the chair.

Dr. Webb, in lieu of a paper, reported a case in obstetrics. Case progressed slowly for four or five hours; was seized with convulsions shortly after the child was born. Gave tinc. veratrum viride, gtts. xx, subcutaneously; pulse soon fell down to 50; emesis was freely induced; very sick at the stomach; followed with alcoholic stimulants. Patient reacted well, and is now convalescing.

Dr. Garrett reported a case in obstetrics. Convulsions setting in with a surprising degree of violence, gave veratrum viride, gtts. vii, and in fifteen minutes gave gtts. v. per-os; reaction followed. Patient, without any untowed symptoms, made a good recovery; generally followed with an active purge.

Dr. Nelson cited a case manifesting the toxical effects of the oil of tansey. Very drowsy, speech incoherent, respirations hurried, heart's action tumultuous. Gave ammonia, digitalis, and brandy. Patient slowly, but effectually, recovered.

Dr. Orr cited a case of fracture of the upper third of the femur, resulting in a compound comminunted fracture; described his mode of bandaging, using wet pasteboards so cut as to fit accurately; knew but little of the clinical history, but learned that it was of an intensely strumous diathesis; had suffered from rachitis, but the case made a satisfactory recovery.

Dr. Stroud thought the bandage as used by Prof. Yandall, known as the plaster-Paris, or spica, bandage, well adapted to such cases.

Dr. Simonton cited a case of incontinence in an adult male, unable to void urine unless in a warm bath, and then his bowels will move; now the case is becoming quite annoying. Gave morphia sulphas, cocaine, and applied fluid extract phytolacca to the prostatic region. The prostate gland eventually abscessed, and, being opened, relief followed.

Dr. Smiley, being a strong advocate of the resolvent properties of phytolacca, held that to be of material benefit, it must be applied in the incipient stages.

Dr. Smith cited a case of gonorrhœal ophthalmia, in which he used solution of corrosive sublimate in conjunction with nitrate of silver, and combined with cocaine, but does not use cocaine *per se*, and held that where the purulent effusion is allowed to accumulate, it would excite inflammation, which often proves destructive to the eye. He insists in strict cleanliness in all affections of the eye and ear.

Dr. White read an excellent paper upon the physiological effects of hyoscin, and especially referred to its hypnotic effect, which is superior to morphia or chloral hydrate.

Dr. Strain cited two cases of labor, each one making apparently an average [?] and thought it rather strange to find in both complete laceration, and, as he stated it, having laced them up, keeping them quiet and bowels quiet for a few days, each did well. He thought there must be some climatic influence favoring rupture, as each one was well developed, having roomy passages.

Dr. Smith read a most excellent paper upon corneal ulceration, and in closing his remarks upon the affections of the eye, said that when a patient calls on a physician for treatment of an eye affection, and he is not entirely satisfied as to his diagnosis, the best application will be found in atropia, 2 grs. to 4 grs. to the drachm of water, with a little cocaine added, four to five times daily, dropped into the inner canthus.

Dr. Nelson cited a case of chancroids, it being impossible to draw down the prepuce. Injecting cocaine he performed circumcission, but after the parts healed, he found a septum had formed above the former, and necessitated a second operation, which resulted satisfactorily.

Dr. Stroud cited a case that had miscarried five times in succession, and asked the opinion of the members as to the probable cause, if any, outside of the artificial habit of the uterus in throwing off its contents at regular periods, which is a recognized fact.

Dr. Dumas said that he had a similar case, and attributed the cause in this case to riding to town every week in a wagon, and thought that where caution was observed, these apparent periodical returns would not occur.

Dr. Inabnit thought habit influenced the genito-urinary passages in a very decided manner.

Dr. Monday thought latent syphilis was more frequently the cause of the various accidents to gestation than almost all other causes combined.

Dr. Nelson cited a case of spinal irritation, and subsequently discovered an accumulation of pus in the thigh, made an incission, evacuated a large secretion, which continued to discharge through a drainage tube.

Dr. Stroud cited a case similar, in which the pus found its way into the peritoneal fold opposite the anterior superior spinous process of the ilium, and from which he withdrew a large quantity of pus.

Dr. Anthony cited the case of a pelvic abscess, the pus contents finding its way to the lungs, and ejected per os.

Dr. Smiley held that where hysterical or choreaic symptoms manifest themselves, we should institute a close examination of the uterus and its apendages.

