# A Correction

**Published:** 1888-09

**Authors:** 


A CORRECTION.—In the proceedings of the *Terrell Medical Society*, published in the last issue of this JOURNAL, Dr. J. H. Smith (of Dallas) is reported as having said: "When a patient calls upon a physician for treatment of an eye affection, aad the physician is not entirely satisfied as to diagnosis, the best application will be found in a solution of atropia, grs. ii to iv, one ounce water, with a little cocaine added, 3 or 4 times daily."

Dr. Smith writes us that the Society reporter misunderstood his meaning, and hence reported his views incorrectly. What Dr. Smith did say was: "When a physician meets with a patient with an *acutely inflamed eye*, accompanied with pain, photophobia, etc., and is in doubt as to diagnosis, *no harm* can come from a solution of atropia, 2 to 4 grs. to the ounce of water."

Dr. Smith adds: "Of course, such treatment would not do for a glaucomatous attack, but this is of such a rare occurrence, compared to other diseases in which an early use of atropia often saves an eye, that it can be excluded from the diagnosis.

"A simple eye affection, as the reporter has it, is a very indefinite term, and might be some lesion in which the atropia solution would be entirely out of place. My remarks were intended for *acute inflammatory* affections, barring glaucoma."

The correction is made at the doctor's request and with pleasure.

